# Socioeconomic Status and Other Related Factors of Seasonal Influenza Vaccination in the South Korean Adult Population Based on a Nationwide Cross-Sectional Study

**DOI:** 10.1371/journal.pone.0117305

**Published:** 2015-02-03

**Authors:** Kyu-Chong Lee, Kyungdo Han, Jin Yong Kim, Ga Eun Nam, Byoung-Duck Han, Koh-Eun Shin, Anna Lee, Byung Joon Ko

**Affiliations:** 1 Department of Radiology, College of Medicine, Korea University, Seoul, South Korea; 2 Department of Biostatistics, College of Medicine, Catholic University, Seoul, South Korea; 3 Department of Neuroscience, College of Medicine, Korea University, Seoul, South Korea; 4 Department of Family Medicine, College of Medicine, Korea University, Seoul, South Korea; 5 Department of Family Medicine, SahmYook Medical Center, Seoul, South Korea; 6 School of Physician Assistant Studies, College of Health Professionals, Pacific University, Hillsboro, Oregon, United States of America; 7 Division of Endocrinology, Diabetes, and Metabolism, Beth Israel Deaconess Medical Center, Harvard Medical School, Boston, Massachusetts, United States of America; 8 Total Healthcare Center, Kangbuk Samsung Hospital, Sungkyunkwan University School of Medicine, Seoul, South Korea; Anhui Medical University, CHINA

## Abstract

**Purpose:**

We investigated the association between seasonal influenza vaccination in South Korea and socioeconomic status (SES) as well as other potential related factors.

**Methods:**

The study was based on data obtained in the Korea National Health and Nutrition Examination Survey from 2010 to 2011. Education level and household income were used as indicators for SES. Univariate and multiple logistic regression analyses were used to evaluate SES and other demographic variables as related factors for influenza vaccination, the primary outcome.

**Results:**

Higher household income was positively associated with higher vaccine uptake in the younger (19–49 years) group [adjusted odds ratio (aOR) 1.55, 95% confidence interval (CI) 1.08–2.23], whereas the low-income and low-education group had increased vaccination coverage than the middle-income and middle-education group in the older (≥ 50 years) group (aOR 1.36, 95% CI 1.09–1.69). Current smokers tend to be unvaccinated in all age groups. Among individuals aged ≥ 50, older age, mild to moderate alcohol consumption, regular exercise, and having co-morbidities were positively associated with vaccination, while those who self-reported their health status as good were less likely to be vaccinated.

**Conclusions:**

The relationship between SES and seasonal influenza vaccination coverage differed between the age groups throughout the adult South Korean population. Public health policies need to address these inequalities.

## Introduction

Influenza is widely known as a serious public health problem that is associated with increased morbidity, mortality, and days of hospitalization, especially in high-risk populations such as the elderly, pregnant women, infants, and those with co-morbidities such as cardiovascular disease, diabetes mellitus, asthma, chronic obstructive pulmonary disease, and malignancy [1, 2].

Numerous previous studies have shown that influenza vaccination is very effective in preventing influenza and safe for humans [3, 4]. Furthermore, influenza vaccination is a cost-effective way to prevent the spread of disease [5, 6]. Based on these studies, the US Centers for Disease Control and Prevention (CDC) has recommended annual influenza vaccination for all persons aged ≥ 6 months since 2010. This represents an expansion of the target populations including all adults aged 19–49 years, which is supported by evidence that influenza vaccine is beneficial to health in all age groups [7]. In South Korea, however, the Korea CDC has recommended annual vaccinations for those at high risk [8]. In an effort to increase the uptake of vaccine, the South Korean government provides the influenza vaccination free of charge at public health centers for the elderly, nursing home residents, and recipients of basic living subsidies. However, other high-risk groups such as pregnant women and people with chronic medical diseases are vaccinated at private clinics at the cost of 15,000 Korean won (14 USD) due to the lack of funding by the national health insurance system. Despite these efforts, not everyone is vaccinated against influenza. According to a previous survey in South Korea, the influenza vaccination coverage in general population was only 34.3% and the rate for those at high risk was 61.3% [9]. Related factors of vaccine uptake included age, co-morbidities, gender, race, and socioeconomic status (SES) [10–12]. Although SES is known to be an important factor associated with influenza vaccination coverage, the trend between SES and vaccination uptake has differed among studies [9, 13–17]. Several studies reported that high SES is positively associated with the uptake of vaccines [13, 14], whereas others did not [9, 16]. A recent report in South Korea showed that lower SES contributed to a higher influenza vaccination coverage; however, the study population was only from an elderly group in a province, which does not represent the population of South Korea [16].

The purpose of this study was to investigate the relationship of SES and potential related factors to the seasonal influenza vaccination national coverage in the South Korean adult population.

## Materials and Methods

### Study Participants

This study was based on the data obtained in the first two years of the Korea National Health and Nutrition Examination Survey (KNHANES) V (2010–2012). KNHANES is a nationwide cross-sectional survey performed to evaluate the health and nutritional status of the general population of South Korea. The survey consists of a health and household interview, nutrition survey, and direct standardized physical examinations. In KNHANES V, rolling survey sampling was adopted and 7,680 households were included. A stratified multistage probability sampling design was used to select the household unit. The samples were weighted to represent all of the South Korean population, taking the complex sampling design with oversampling and survey non-response into consideration. Initially, 17,476 participants were enrolled. Those younger than 19 years of age (*n* = 4,170) were excluded and 1,169 participants were excluded due to missing values of variables of seasonal influenza vaccination, education level, and household income. Finally, 12,137 participants were included in this study. All participants of the survey provided written informed consents and the Institutional Review Board of the the Korea Centers for Disease Control and Prevention approved the study protocol.

### Description of Demographic Variables

The information on the seasonal influenza vaccination status in the previous 12 months and other demographic variables was collected at the health interview.

The study populations were divided into two age groups: ages between 19 and 50 and ages 50 years and older. The cut-off point for dividing age groups was determined at the age of 50 rather than 65 based on a previous study [18] and after calculating interaction between age groups and SES levels. Education level and household income were used as indicators for SES. Level of education was classified into four groups: elementary school or lower, middle school, high school, and college and higher. Education levels were further divided into low (≤ elementary school), middle (middle and high school), and high (≥ college). Household income was divided into four groups by quartiles and then defined as low (Q1), middle (Q2 + Q3), and high (Q4), accordingly. Participation status of the program conducted by the National Basic Livelihood Security System was divided into three groups: current, previous, and non-participating. Residential area was divided into two areas: rural and urban. Marital status was divided into three groups: married, single, and divorced/separated/widowed. Smoking status was classified into three groups: non-smokers, ex-smokers, and current smokers. Those who smoked more than five packs in a lifetime were defined as smokers. We distinguished ex-smokers from current smokers based on their present smoking status. Alcohol consumption was divided into three groups based on the frequency of monthly drinking: non-drinkers, mild to moderate drinkers, and heavy drinkers. Those who drank more than once a month were defined as drinkers, and those who drank more than 20 grams of alcohol a day were defined as heavy drinkers. Physical activity was divided into two groups: non-regular exercise and regular exercise. Regular exercise was defined as exercising more than three times a week and more than 20 minutes at a time. Self-report of health status was divided into three groups: poor, moderate, and good. Those who answered “yes” to the question regarding sustained depressed mood for at least two consecutive weeks were regarded as having depression symptom. The presence of co-morbidities was determined by asking the respondents if they were ever diagnosed with any of the following: cardiovascular diseases such as angina and myocardial infarction, diabetes mellitus, asthma, chronic liver diseases such as liver cirrhosis and chronic hepatitis, stroke, and malignancy.

### Statistical Analyses

The main outcome was seasonal influenza vaccination coverage. General characteristics according to influenza vaccination status are presented as numbers with percentages. We used both univariate and multiple logistic regression analyses to evaluate SES and other demographic variables as related factors for influenza vaccination. The interaction of education level and household income for both gender and age group (gender*education, gender*income, age group*education, and age group*income, respectively) was calculated before fitting the multiple logistic regression model as a covariate. In the multiple logistic regression analyses, we entered age, gender, education level, household income, and other demographic variables into the model. Additionally, odds ratios (ORs) and 95% confidence intervals (CIs) for receiving influenza vaccine according to combinations of SES (low-income and low-education group; high-income and high-education group) were calculated. The middle-income and middle-education group served as a reference and the model was adjusted for age and gender. All analyses were conducted using SAS (Version 9.2; SAS Institute, Cary, NC, USA) and weighted to allow for oversampling, non-response, and the Korean population in 2010–2011. A p value <0.05 was considered statistically significant.

## Results

Table 1 shows the influenza vaccination coverage according to the demographic factors between the age groups. People aged 19 to 49 (the younger group) achieved a vaccination prevalence of 20.9%, whereas 47.9% of those aged 50 and older (the older group) were vaccinated. Overall, in both age groups, immunization was lower in men (26.5%) than women (35.5%).

**Table 1:** The influenza vaccination coverage according to the demographic factors.

	**Total**			**19 ≤ Age < 50**			**Age ≥ 50**		
	**All, *n* (*n* = 12,137)**	**Vaccinated, *n* (%^a^)** **(*n* = 4,673)**	**P value**	**All, *n* (*n* = 5,944)**	**Vaccinated**, ***n* (%^a^) (*n* = 1,354)**	**P value**	**All, *n* (*n* = 6,193)**	**Vaccinated, *n* (%^a^)** **(*n* = 3,319)**	**P value**
**Age group**			<0.001						
19–49 years	5,944	1,354 (20.9)		-	-		-	-	
≥ 50 years	6,193	3,319 (47.9)		-	-		-	-	
**Gender**			<0.001			<0.001			<0.001
Male	5,204	1,833 (26.5)		2,516	484 (17.9)		2,688	1,349 (42.2)	
Female	6,933	2,840 (35.5)		3,428	870 (24.0)		3,505	1,970 (52.9)	
**Level of education**			<0.001			0.004			<0.001
Elementary school or lower	3,118	1,974 (57.3)		114	31 (24.0)		3,004	1,943 (60.1)	
Middle school	1,337	559 (35.5)		259	55 (20.8)		1,078	504 (42.1)	
High school	4,025	1,122 (22.4)		2,605	514 (18.4)		1,420	608 (35.6)	
College and higher	3,657	1,018 (24.4)		2,966	754 (23.3)		691	264 (31.9)	
**Household income**			<0.001			0.006			<0.001
Lowest	3,033	1,739 (45.9)		553	92 (15.2)		2,480	1,647 (62.1)	
Medium-lowest	2,787	1,045 (29.1)		1,449	329 (20.3)		1,338	716 (46.7)	
Medium-highest	3,222	968 (26.2)		2,025	461 (21.3)		1,197	507 (38.6)	
Highest	3,095	921 (26.3)		1,917	472 (23.2)		1,178	449 (33.6)	
**Participation of the program under the National Basic Livelihood Security System**			<0.001			0.830			<0.001
Non-participating	11,534	4,376 (30.6)		5,736	1,302 (20.8)		5,798	3,074 (47.2)	
Previous	304	130 (34.0)		116	28 (23.1)		188	102 (49.5)	
Current	296	165 (44.5)		92	24 (22.2)		204	141 (65.0)	
Missing	3	2 (76.2)		0	0		3	2 (76.2)	
**Residency area**			<0.001			0.278			<0.001
Urban	9,573	3,466 (29.4)		5,130	1,183 (21.3)		4,443	2,283 (45.3)	
Rural	2,564	1,207 (37.6)		814	171 (18.8)		1,750	1,036 (54.4)	
**Marital status**			<0.001			<0.001			<0.001
Married	9,049	3,512 (32.8)		4,180	1,063 (24.2)		4,869	2,449 (44.3)	
Divorced/separated/widowed	1,451	890 (54.1)		179	41 (19.2)		1,272	849 (63.1)	
Single	838	120 (13.8)		823	118 (13.9)		15	2 (6.5)	
Missing	799	151 (16.5)		762	132 (16.0)		37	19 (33.5)	
**Smoking status**			<0.001			<0.001			<0.001
Non-smoker	7,358	2,965 (34.3)		3,664	918 (23.7)		3,694	2,047 (51.7)	
Ex-smoker	1,208	517 (33.1)		452	108 (20.8)		756	409 (46.0)	
Current smoker	2,438	649 (21.1)		1,455	243 (15.9)		983	406 (34.2)	
Missing	1,133	542 (37.5)		373	85 (19.6)		760	457 (54.2)	
**Alcohol consumption**			<0.001			0.030			<0.001
Non-drinker	3,336	1,739 (43.5)		892	225 (22.6)		2,444	1,514 (56.5)	
Mild to moderate drinker	6,815	2,400 (29.1)		3,936	930 (21.6)		2,879	1,470 (46.8)	
Heavy drinker	1,889	501 (22.4)		1,059	185 (17.4)		830	316 (32.3)	
Missing	97	33 (32.1)		57	14 (23.9)		40	19 (50.1)	
**Physical activity**			0.124			0.783			0.179
Non-regular exercise	9,738	3,789 (31.4)		4,760	1,094 (21.0)		4,978	2,695 (48.3)	
Regular exercise	2,367	862 (29.5)		1,117	259 (20.6)		1,190	603 (45.5)	
Missing	32	22 (65.9)		7	1 (18.0)		25	21 (81.8)	
**Self-report of health status**			<0.001			0.040			<0.001
Poor	2,411	1,224 (41.8)		716	154 (20.4)		1,695	1,070 (58.2)	
Moderate	5,537	2,052 (30.5)		2,905	702 (22.5)		2,632	1,350 (45.6)	
Good	4,189	1,397 (26.5)		2,323	498 (19.0)		1,866	899 (42.2)	
**Depression symptom**			0.086			0.566			0.415
No	10,489	3,997 (30.6)		5,274	1,216 (21.0)		5,215	2,781 (47.5)	
Yes	1,626	659 (33.3)		667	137 (19.9)		959	522 (49.2)	
Missing	22	17 (86.1)		3	1 (53.6)		19	16 (90.8)	
**Any co-morbidities^b^**			<0.001			0.301			<0.001
No	9,636	3,287 (27.9)		5,433	1,223 (20.7)		4,203	2,064 (43.8)	
Yes	2,501	1,386 (46.2)		511	131 (22.9)		1,990	1,255 (57.2)	
**Cardiovascular disease^c^**			<0.001			<0.001			<0.001
No	11,799	4,446 (30.5)		5,928	1,353 (20.9)		5,871	3,093 (47.2)	
Yes	337	226 (58.4)		16	1 (1.8)		321	225 (63.1)	
Missing	1	1 (100.0)		0	0		1	1 (100.0)	
**Diabetes mellitus**			<0.001			0.948			<0.001
No	10,874	3,963 (29.6)		5,750	1,311 (20.9)		5,124	2,652 (46.2)	
Yes	1,263	710 (47.3)		194	43 (20.7)		1,069	667 (56.3)	
**Asthma**			<0.001			0.050			<0.001
No	11,723	4,427 (30.5)		5,810	1,310 (20.7)		5,913	3,117 (47.0)	
Yes	414	246 (48.3)		134	44 (28.2)		280	202 (68.0)	
**Chronic liver disease^d^**			0.574			0.335			0.431
No	11,906	4,579 (31.1)		5,852	1,336 (21.0)		6,054	3,243 (48.0)	
Yes	231	94 (29.1)		92	18 (16.3)		139	76 (44.0)	
**Stroke**			<0.001			0.644			<0.001
No	11,894	4,510 (30.6)		5,934	1,352 (20.9)		5,960	3,158 (47.3)	
Yes	242	162 (61.9)		10	2 (15.4)		232	160 (65.3)	
Missing	1	1 (100.0)		0	0		1	1 (100.0)	
**Malignancy**			<0.001			0.176			0.002
No	11,708	4,439 (30.6)		5,855	1,328 (20.8)		5,853	3,111 (47.4)	
Yes	429	234 (49.1)		89	26 (28.0)		340	208 (58.9)	

^a^Weighted data.

^b^Subjects who had one or more co-morbidities including cardiovascular disease, diabetes, asthma, chronic liver disease, stroke, and malignancy.

^c^Angina, myocardial infarction.

^d^Liver cirrhosis, chronic hepatitis

There was no interaction of education level (p = 0.977) and household income (p = 0.920) for gender. There was no interaction of education level (p = 0.150) but a significant household income interaction (p = 0.017) for age groups.

Vaccination in both age groups showed a clear difference according to their social class (Table 2). Stepwise increment in education level corresponded to a decrease in vaccination prevalence in the older group (p for trend < 0.001); however, the relationship was not significant in multivariable model. Among the younger individuals, those with the highest household income were more likely to get vaccinated [adjusted OR (aOR) 1.55, 95% CI 1.08–2.23, p for trend = 0.003] than those with lower incomes, whereas the opposite was true among older subjects in the univariate analysis (OR 0.33, 95% CI 0.26–0.40, p for trend < 0.001) though the association lost its significance after adjusting for other covariates.

**Table 2:** Factors associated with seasonal influenza vaccine uptakes among South Korean adults, stratified by age groups.

	**19 ≤ Age < 50**		**Age ≥ 50**	
	**Univariate**	**Multivariate**	**Univariate**	**Multivariate**
**Age** (∆ 1 yr)	1.01 (1.00–1.02)	0.97 (0.95–0.98)	1.12 (1.11–1.13)	1.12 (1.10–1.13)
**Gender**				
Male	1.00	1.00	1.00	1.00
Female	1.49 (1.28–1.74)	1.13 (0.90–1.42)	1.75 (1.51–2.02)	1.23 (0.96–1.57)
**Level of education**				
Elementary school or lower	1.00	1.00	1.00	1.00
Middle school	0.84 (0.46–1.56)	0.82 (0.43–1.56)	0.48 (0.40–0.57)	1.13 (0.90–1.41)
High school	0.70 (0.42–1.15)	0.68 (0.40–1.15)	0.35 (0.29–0.42)	0.92 (0.73–1.15)
College and higher	0.93 (0.57–1.53)	0.81 (0.47–1.39)	0.32 (0.25–0.41)	0.88 (0.65–1.19)
P for trend	0.106	0.654	< 0.001	0.272
**Household income**				
Lowest	1.00	1.00	1.00	1.00
Medium-lowest	1.34 (0.95–1.88)	1.15 (0.78–1.68)	0.55 (0.45–0.67)	1.06 (0.86–1.30)
Medium-highest	1.47 (1.05–2.05)	1.29 (0.88–1.89)	0.40 (0.33–0.49)	0.94 (0.76–1.17)
Highest	1.67 (1.21–2.30)	1.55 (1.08–2.23)	0.33 (0.26–0.40)	0.84 (0.64–1.09)
P for trend	< 0.001	0.003	< 0.001	0.144
**Participation of the program. under the National Basic Livelihood Security System**				
Non-participating	1.00	1.00	1.00	1.00
Previous	1.24 (0.77–2.00)	1.27 (0.73–2.18)	1.03 (0.71–1.49)	0.61 (0.37–1.02)
Current	1.16 (0.65–2.08)	1.49 (0.80–2.79)	2.07 (1.38–3.10)	1.51 (0.98–2.34)
**Residency area**				
Urban	1.00	1.00	1.00	1.00
Rural	0.88 (0.66–1.16)	0.85 (0.62–1.17)	1.39 (1.16–1.67)	1.16 (0.94–1.41)
**Marital status**				
Married	1.00	1.00	1.00	1.00
Divorced/separated/widowed	0.75 (0.50–1.13)	0.91 (0.60–1.40)	2.23 (1.89–2.63)	0.86 (0.71–1.06)
Single	0.50 (0.39–0.64)	0.34 (0.24–0.46)	0.09 (0.02–0.52)	0.15 (0.03–0.75)
**Smoking status**				
Non-smoker	1.00	1.00	1.00	1.00
Ex-smoker	0.86 (0.66–1.10)	0.94 (0.71–1.26)	0.81 (0.66–0.98)	0.83 (0.63–1.10)
Current smoker	0.60 (0.51–0.71)	0.72 (0.56–0.94)	0.48 (0.40–0.58)	0.68 (0.52–0.89)
**Alcohol consumption**				
Non-drinker	1.00	1.00	1.00	1.00
Mild to moderate drinker	1.00 (0.79–1.26)	1.15 (0.90–1.46)	0.67 (0.58–0.78)	1.24 (1.05–1.46)
Heavy drinker	0.72 (0.54–0.96)	0.95 (0.68–1.33)	0.35 (0.28–0.43)	0.93 (0.70–1.23)
**Physical activity**				
Non-regular exercise	1.00	1.00	1.00	1.00
Regular exercise	1.06 (0.87–1.28)	1.18 (0.97–1.43)	0.93 (0.78–1.10)	1.28 (1.04–1.56)
**Self-report of health status**				
Poor	1.00	1.00	1.00	1.00
Moderate	1.09 (0.85–1.39)	1.18 (0.91–1.53)	0.59 (0.51–0.68)	0.91 (0.76–1.09)
Good	0.90 (0.70–1.14)	0.89 (0.69–1.14)	0.51 (0.43–0.61)	0.74 (0.60–0.90)
**Depression symptom**				
No	1.00	1.00	1.00	1.00
Yes	0.91 (0.71–1.16)	0.86 (0.66–1.12)	1.08 (0.91–1.29)	0.93 (0.76–1.14)
**Any co-morbidities**				
No	1.00	1.00	1.00	1.00
Yes	1.15 (0.89–1.48)	1.28 (0.96–1.69)	1.63 (1.42–1.88)	1.31 (1.10–1.56)

All values are odds ratios with 95% confidence intervals or P for trend.

P value for interaction between age group*household income = 0.017.

Unmarried individuals tended to be unvaccinated than those who were married in all age groups (aOR 0.34, 95% CI 0.24–0.46 in the younger group; aOR 0.15, 95% CI 0.03–0.75 in the older group). Current smokers were less likely to be vaccinated than non-smokers (aOR 0.72. 95% CI 0.56–0.94 in the younger group; aOR 0.68, 95% CI 0.52–0.89 in the older group).

Among those aged ≥ 50, older age (aOR 1.12, 95% CI 1.10–1.13), mild to moderate alcohol consumption (aOR 1.24, 95% CI 1.05–1.46), regular exercise (aOR 1.28, 95% CI 1.04–1.56), and having co-morbidities (aOR 1.31, 95% CI 1.10–1.56) were positively associated with vaccination uptake, while those who self-reported their health status as good were less likely to be vaccinated than those who reported as poor (aOR 0.74, 95% CI 0.60–0.90).

**Fig 1:**
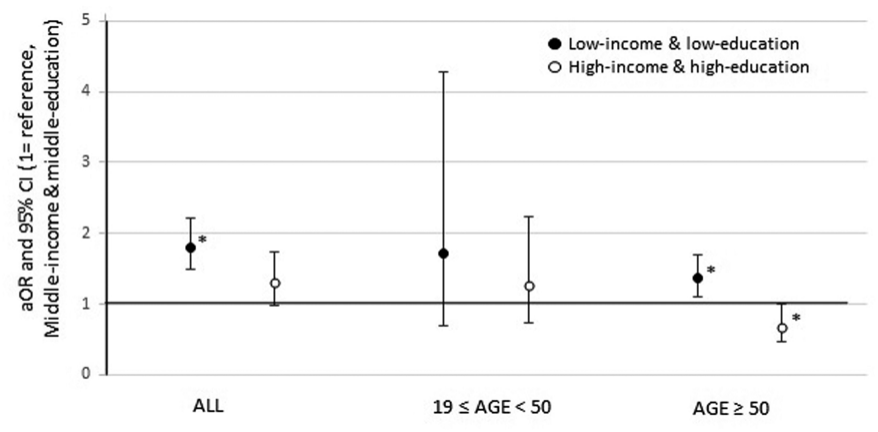
Adjusted OR and 95% CI for influenza vaccination by education and income in age groups. Logistic regression model included age and gender. Household income was divided by quartiles and defined as low (Q1), middle (Q2 + Q3), and high (Q4), accordingly. Education levels were divided into low (≤ elementary school), middle (middle and high school), and high (≥ college). Middle income and middle education group served as the reference. OR, odds ratio; CI, confidence interval. *P < 0.05.

Fig. 1 exhibits the joint effect of education level and household income on seasonal influenza vaccination uptake in age groups. After adjusting for gender and age, the low-income and low-education group had a higher vaccination coverage (aOR 1.36, 95% CI 1.09–1.69) than the middle-income and middle-education group (reference group), whereas the high-income and high-education group showed a lower vaccination uptake (aOR 0.68, 95% CI 0.46–0.99) than the reference group among subjects aged ≥ 50.

## Discussion

This study demonstrated a different relationship between SES and the seasonal influenza vaccination coverage according to the age groups in the South Korean adult population. In the younger group, aged 19 to 49, the vaccination uptake increased with household income, while the low SES group tended to be vaccinated more in the older group, aged ≥ 50.

Previous studies of the effects of SES on the influenza vaccination coverage showed inconsistent results. An international observational study reported that lower SES was associated with lower influenza vaccination coverage [19]. A study in Italy showed that lower SES measured by education and occupational level was related to lower influenza vaccination uptake; however, among adults aged 25 to 44 years, there was a reverse J-shaped relationship between the education level and influenza vaccination uptake [14]. A study in the United States showed that the vaccination coverage according to SES was different in the two age groups [18]. According to this study, among those aged older than 50 years, those with a lower SES had a higher influenza vaccination coverage; however, among those younger than 50 years of age, those with a higher SES had a higher influenza vaccination uptake. In contrast, other investigators have reported that lower SES was associated with higher influenza vaccination uptake in South Korea [9, 16] and in China [20]. When the association between SES and influenza vaccination uptake is examined, the free vaccination policy to a vulnerable population in a country should be considered because people with low SES may be vaccinated more due to the policy. The requirements of personal co-payment for vaccination could be a barrier to vaccination and may reduce the prevalence of immunization [20–23]. The proportion of vaccination was nearly double for those eligible for free vaccination compared to those paying a fee in South Korea in the 2009–2010 novel influenza A (H1N1) season [21].

We found a socioeconomical difference in vaccination between age groups, which is parallel to a previous study in the United States [18]. There are several reasons why the differences exist. Those who have a higher education level and/or household income are more likely to receive preventive health services because they may have more knowledge about health-related services and the effectiveness of preventive medicines [25–28]. Furthermore, they could afford vaccination and these factors may contribute to the positive association between household income and influenza vaccination uptake in the younger individuals. On the other hand, the older people aged ≥ 50 had lower SES, as shown in Table 1, and a substantial proportion (36.7%) were aged ≥ 65 who could have received influenza vaccination free of charge at public health centers, which could account to the finding that low SES was associated with high vaccination prevalence in older people. The finding of this study implies that public health authorities should focus separately on the target groups according to age groups to improve seasonal influenza vaccination coverage.

This study included several demographic factors that can act as confounding factors based on previous studies. In accordance with previous reports, we demonstrated that among the people aged ≥ 50, older age [16, 29, 30], mild to moderated alcohol consumption [16, 25], and regular exercise [16, 25] were positive predictors for a higher vaccination uptake. Furthermore, our study suggests that fewer current smokers were vaccinated than non-smokers in all age groups, which is consistent with earlier studies [29, 30]. Those who exhibit a healthy lifestyle such as mild to moderate alcohol consumption, regular exercise, and/or non-smoking may be more likely to pursue general health-protective behaviors or health-preventive care, and thus they have more opportunities to be vaccinated [9, 25]. Single marital status was a predictor of lower vaccination uptake in all the populations, which implies the importance of social supports as well as a greater likelihood of having previously established a regular source of health care found in married couples [31]. However, the number of unmarried individuals was small in the people aged ≥ 50 (15 out of 6,193 people), which requires a careful interpretation of the results. This study suggests that in older adults, fewer participants who self-reported their health status as good tended to be vaccinated than those who self-reported their health status as poor. Those who self-reported their health status as poor may be more concerned about their health, thus are more likely to visit health care providers and given more opportunities to be vaccinated [16] or they may have poor health such as co-morbidities (e.g. asthma), which requires immunization. Finally, in accordance with previous studies, older adults with co-morbidities were vaccinated more than those without diseases [9, 16, 25, 27, 30].

The low vaccination coverage of current smokers should be underscored because smoking is an important risk factor for complications related to influenza infection [32]. Vaccination promotion strategy for smokers as well as at-risk groups may be concerned for future vaccination programs. The cost of receiving a seasonal influenza vaccine may be driving the relatively lower vaccination coverage in high-risk groups. Public health authorities not only have to more actively publicize vaccination but also should consider expansion of free vaccination policy and encouraging health professionals to recommend vaccination to these groups because these approaches have been known to increase influenza vaccination coverage [21, 22, 24, 33].

To our knowledge, this is the first report to clarify the relationship between SES and influenza vaccination coverage in the South Korean adult population using nationally representative data. However, this study had several limitations. First, the design of the study was cross-sectional and retrospective. Thus, the results could not imply a causal relationship between SES and influenza vaccination uptake. Second, we did not include all confounding factors based on previous studies, such as beliefs about influenza vaccination and vaccination in the previous season. Third, high-risk groups in this study only consisted of people with co-morbidities and the elderly, and did not include other groups such as pregnant women. Finally, the information on the vaccination uptake as well as other demographic variables including co-morbidities was collected by health interview, not by chart review, thus recall bias may occur. However, some reports have demonstrated that the sensitivity and specificity of self-reported influenza vaccination data are respectively, high and moderate compared to chart reviews [34, 35].

In conclusion, low household income is associated with poor vaccination coverage in the younger population, whereas the low-income and low-education group has higher vaccine uptake in the individuals aged ≥ 50. Furthermore, vaccination coverage is poor in current smokers. Public health policies in South Korea need to address these inequalities. To supplement this study, further studies with a prospective design and including other related factors are warranted.
